# The General Movement Assessment Helps Us to Identify Preterm Infants at Risk for Cognitive Dysfunction

**DOI:** 10.3389/fpsyg.2016.00406

**Published:** 2016-03-22

**Authors:** Christa Einspieler, Arend F. Bos, Melissa E. Libertus, Peter B. Marschik

**Affiliations:** ^1^Research Unit iDN – interdisciplinary Developmental Neuroscience, Institute of Physiology, Center for Physiological Medicine, Medical University of GrazGraz, Austria; ^2^Division of Neonatology, Department of Pediatrics, Beatrix Children’s Hospital, University Medical Center, University of GroningenGroningen, Netherlands; ^3^Department of Psychology, Learning Research Development Center, University of PittsburghPittsburgh, PA, USA; ^4^Center of Neurodevelopmental Disorders, Department of Women’s Children’s Health, Karolinska InstitutetStockholm, Sweden

**Keywords:** cognition, fidgety movements, general movements, intelligence, motor behavior, posture, preterm

## Abstract

Apart from motor and behavioral dysfunctions, deficits in cognitive skills are among the well-documented sequelae of preterm birth. However, early identification of infants at risk for poor cognition is still a challenge, as no clear association between pathological findings based on neuroimaging scans and cognitive functions have been detected as yet. The Prechtl General Movement Assessment (GMA) has shown its merits for the evaluation of the integrity of the young nervous system. It is a reliable tool for identifying infants at risk for neuromotor deficits. Recent studies on preterm infants demonstrate that abnormal general movements (GMs) also reflect impairments of brain areas involved in cognitive development. The aim of this systematic review was to discuss studies that included (i) the Prechtl GMA applied in preterm infants, and (ii) cognitive outcome measures in six data bases. Seven studies met the inclusion criteria and yielded the following results: (a) children born preterm with consistently abnormal GMs up to 8 weeks after term had lower intelligence quotients at school age than children with an early normalization of GMs; (b) from 3 to 5 months after term, several qualitative, and quantitative aspects of the concurrent motor repertoire, including postural patterns, were predictive of intelligence at 7–10 years of age. These findings in 428 individuals born preterm suggest that normal GMs along with a normal motor repertoire during the first months after term are markers for normal cognitive development until at least age 10.

## Introduction

Children born preterm have higher rates of adverse motor, cognitive, behavioral, and psychiatric outcomes than their term-born peers, even in the absence of brain injury (e.g., Johnson, 2007; Doyle and Anderson, 2010; Bos and Roze, 2011; Johnson et al., 2015). Cognitive deficits occur in 25–50% of children born preterm, especially if their birth weight is under 1500 g (Bhutta et al., 2002; Johnson, 2007; Saigal and Doyle, 2008). On average, children born at <32 weeks’ gestation score at least 11 points below their term-born peers in a cognitive test (Foulder-Hughes and Cooke, 2003; Larroque et al., 2005). This number increases with inverse proportion to each week of shorter gestation (Bhutta et al., 2002; Johnson, 2007). Although the age of cognitive evaluation had no significant impact on the reported differences (Bhutta et al., 2002), the deficit usually becomes more evident when children enter school and face higher cognitive demands.

The identification of infants at risk for poor cognition is a challenging issue. Clear associations between alterations in the brain structure and cognitive deficits are still scarce, although global white matter damage is common among children born preterm, and gray matter volumes are diminished (Nosarti et al., 2008; Soria-Pastor et al., 2008; Volpe, 2009; Ullman et al., 2015). Recently, de Vries et al. (2015) suggested that subtle white matter injuries and cerebellar lesions may be specifically associated with cognitive problems. Advanced brain imaging techniques such as tract-based special statistics of diffusion tensor imaging are suggested to potentially define early markers for cognitive development in preterm infants if applied at term-equivalent age (van Kooij et al., 2015).

These neuroimaging techniques are not globally available and do not supersede the need to functionally assess the integrity of the young nervous system. One of the most reliable and sensitive non-intrusive techniques, which has been used for more than 25 years, is the assessment of GMs, an early spontaneous movement pattern (Prechtl, 1997, 2001; Prechtl et al., 1997; Einspieler and Prechtl, 2005; Bosanquet et al., 2013).

## What are General Movements?

Without even being constantly triggered by a specific sensory input, the fetal and neonatal nervous system generates a variety of motor patterns such as simple startles or twitches, but also more complex patterns such as stretching, yawning or GMs (Einspieler et al., 2008, 2012c). The latter involve the entire body in a variable sequence of neck, arm, trunk, and leg movements. They wax and wane, varying in intensity, speed, and range of motion, and have a gradual onset and end. Rotations around the limb axes and slight changes in the direction of movement create the impression of fluency and elegance (Prechtl, 1990; Einspieler and Prechtl, 2005). By and large, GMs have a similar appearance from early fetal life until the end of the second month after term; from term age onward, they are called “writhing movements.” At 6–9 weeks postterm age, writhing movements gradually disappear and GMs of a fidgety character gradually emerge (Einspieler and Prechtl, 2005). Observable from 3 to 5 months after term, so-called “fidgety movements” are tiny movements of the neck, trunk, and limbs in all directions and of variable acceleration (Prechtl et al., 1997).

General movements are generated by a neural network, the central pattern generators (CPGs), which are most likely located in the brainstem. In order to lend variability to the motor output, supraspinal projections activate, inhibit and, most importantly, modulate the CPG activity, as does the sensory feedback (Einspieler et al., 2004; Einspieler and Marschik, 2012).

Reduced modulation of the CPGs results in less variable (i.e., abnormal) movements and indicates fetal or neonatal compromise. Abnormal GMs during preterm and term age are classified as (a) poor repertoire GMs, whereby the sequence of movement components is monotonous and the intensity, speed, and range of motion lack the normal variability; (b) cramped-synchronized GMs, which lack the usual smoothness and fluency and appear rigid as the limb and trunk muscles contract almost simultaneously and relax almost simultaneously; (c) chaotic GMs, which are abrupt and tremulous, of large amplitude and high speed; they rarely occur at term age or beyond, but are typically observed in the moderate preterm age (Ferrari et al., 1990; Bos et al., 1997; Einspieler and Prechtl, 2005; Einspieler et al., 2015a). Abnormal fidgety movements are exaggerated in amplitude, speed, and jerkiness (Prechtl et al., 1997). If fidgety movements are only sporadically present or altogether absent at 3–5 months, the infant is likely to develop severe neurological deficits such as cerebral palsy (e.g., Prechtl et al., 1997; Einspieler and Prechtl, 2005; Bruggink et al., 2009; Yang et al., 2012; Bosanquet et al., 2013; Einspieler et al., 2015b).

Apart from incipient yet promising and time-consuming attempts to analyze GMs with the aid of computer-based tools (e.g., Adde et al., 2009; Einspieler and Marschik, 2013; Marcroft et al., 2015), the state-of-the-art GMA applied in research and clinical routine is based on visual Gestalt perception of age-specific normal and abnormal patterns. Experienced observers consistently achieved high inter-scorer agreements ranging from Kappa 0.85 to 0.94 (e.g., Einspieler and Prechtl, 2005; Valentin et al., 2005; Mutlu et al., 2008). For this standardized assessment, the comfortably dressed infant is video-taped in supine position for 3–5 min, provided that the infant is not fussy or crying (Einspieler et al., 1997). In other words, GMA is non-invasive, non-intrusive, cost-effective, and highly reliable.

Since its introduction 26 years ago (Prechtl, 1990), GMA has been increasingly appreciated for predicting motor dysfunctions, especially cerebral palsy (for reviews, see e.g., Spittle et al., 2008; Burger and Louw, 2009; Einspieler et al., 2012b; Noble and Boyd, 2012; Bosanquet et al., 2013). Cramped-synchronized GMs and the absence of fidgety movements are particularly good predictors of cerebral palsy (e.g., Prechtl et al., 1997; Einspieler et al., 2012b), whereas poor repertoire GMs and abnormal fidgety movements tend to be associated with minor neurological dysfunctions (e.g., Nakajima et al., 2006; Einspieler et al., 2007; Bruggink et al., 2008; Yuge et al., 2011). Only recently was the focus put on the question whether or not GMA might also shed light on cognitive and language development (e.g., Butcher et al., 2009; Bruggink et al., 2010; Spittle et al., 2013) or behavioral, mental, and genetic disorders (e.g., Hadders-Algra et al., 2009; Marschik et al., 2009, 2015; Einspieler et al., 2012a, 2014; Zappella et al., 2015).

## Method

Our aim was to systematically review studies that included cognitive outcome assessments of children born preterm whose GMs had been examined during infancy. A comprehensive literature search was performed using the following databases: Medline, CINAHL, The Cochrane Library, Science Direct, PsycINFO, and EMBASE. The search strategy included the MeSH terms and search strings (‘GM*’ OR ‘spontaneous motor activity’) AND (‘cognition’ OR ‘cognitive’ OR ‘intelligence’). Requests were limited to human participants. The search strategy additionally included studies published on the website of the General Movement Trust (www.general-movements-trust.info). Furthermore, we searched for alternative studies carried out by the authors of the publications picked for this review, using the same search strategies. The following inclusion criteria were applied: (a) primary research based on the Prechtl assessment of GMs; (b) study samples consisting of preterm infants; (c) the outcome had to be assessed at 12 months postterm age or later. Seven studies met these criteria, comprising a total of 428 individuals; the cognitive outcomes were assessed at ages ranging from 2 to 11 years; details are provided in **Tables 1** and **2**. The studies were conducted in Australia (Spittle et al., 2013), Italy (Beccaria et al., 2012), Norway (Fjørtoft et al., 2013; Grunewaldt et al., 2014), Slovenia (Kodric et al., 2010), and the Netherlands (Butcher et al., 2009; Bruggink et al., 2010). As GMs occur in age-specific patterns we present our results separately for studies involving GMA up to the end of the first month post term (writhing GMs; Section “The Link between Early Normalization of GMs and Cognitive Development”) and studies focusing on GMA from 3 to 5 months postterm age (fidgety movements; Section “Fidgety Movements May Not Predict Cognitive Development, But Concurrent Movements and Postures Do”).

**Table 1:** Studies associating writhing GMs with cognitive development, listed according to the age of outcome assessment.

Reference	Cohort	Outcome assessment	Cut-off points	Predictive values
Beccaria et al., 2012	*N* = 79born ≤ 32 weeks’ gestation	**2 years** Griffiths Scales of Mental Development, Italian version	Not given	Not given
Spittle et al., 2013	*N* = 94born < 30 weeks’ gestation	**2 years** Bayley Scales of Infant and Toddler Development, third edition	Abnormal vs. normal writhing GMs related to moderate to severe cognitive impairment	Sensitivity: 80%(95% CI: 44–96%)Specificity: 41%(95% CI: 31–53%)PPV: 14%(95% CI: 7–27%)NPV: 94%(95% CI: 80–99%)Accuracy: 46%(95% CI: 36–56%)
Kodric et al., 2010	*N* = 26born < 36 weeks’ gestation	**2–3 years** (mean = 28.3 months; *SD* = 5.6 months)Bayley Scales of Infant Development, second edition, Slovenian version	Abnormal vs. normal writhing GMs related to *MDI* ≤ 84 vs. *MDI* > 84(excluding *n* = 3 with cerebral palsy)	Sensitivity: 100%Specificity: 28%
Spittle et al., 2013	*N* = 85born < 30 weeks’ gestation	**4 years** Differential Ability Scale, second edition	Abnormal vs. normal writhing GMs related to moderate to severe impairment in the general cognitive ability	Sensitivity: 89%(95% CI: 64–98%)Specificity: 48%(95% CI: 36–61%)PPV: 33%(95% CI: 20–48%)NPV: 67%(95% CI: 52–80%)Accuracy: 57%(95% CI: 46–68%)
Bruggink et al., 2010	*N* = 60born < 34 weeks’ gestation	**7–11 years** (median = 9 years)Wechsler Intelligence Scale for Children-III, Dutch version	Consistently abnormal GMs to 8 weeks after term vs. normal GMs before 8 weeks after termrelated to total *IQ* < 85 vs. total *IQ* ≥ 85	Sensitivity: 67%(95% CI: 43–91%)Specificity: 71%(95% CI: 58–84%)PPV: 43%(95% CI: 23–63%)NPV: 86%(95% CI: 75–97%)

*CI, confidence interval; IQ, intelligence quotient; MDI, mental developmental index; NPV, negative predictive value; PPV, positive predictive value; SD, standard deviation.*

**Table 2:** Assessment of fidgety movements and the concurrent motor repertoire (at 3–5 months after term) and its predictive value for cognitive development (listed according to the age of outcome assessment).

Reference	Cohort	Outcome assessment	Cut-off points	Predictive values
Spittle et al., 2013	*N* = 94born < 30 weeks’ gestation	**2 years** Bayley Scales of Infant and Toddler Development, third edition	Abnormal/absent fidgety movements vs. normal fidgety movements related to moderate to severe cognitive impairment	Sensitivity: 70%(95% CI: 35–92%)Specificity: 85%(95% CI: 75–91%)PPV: 35%(95% CI: 16–59%)NPV: 96%(95% CI: 88–99%)Accuracy: 83%(95% CI: 75–91%)
Spittle et al., 2013	*N* = 85born < 30 weeks’ gestation	**4 years** Differential Ability Scale, second edition	Abnormal/absent fidgety movements vs. normal fidgety movements related to moderate to severe impairment of the general cognitive ability	Sensitivity: 42%(95% CI: 21–66%)Specificity: 88%(95% CI: 77–94%)PPV: 50%(95% CI: 26–74%)NPV: 84%(95% CI: 72–91%)Accuracy: 77%(95% CI: 68–86%)
Butcher et al., 2009	*N* = 65born < 34 weeks’ gestation	**7–11 years** (median = 9 years)Wechsler Intelligence Scale for Children-III, Dutch version	Not applicable	Not given
Fjørtoft et al., 2013	*N* = 40*n* = 31 with very low birth weight, mean gestational age = 26.8 weeks (*SD* = 1.9) *n* = 9 born at term (neonatal encephalopathy, intracerebral abscess)	**10 years** Wechsler Intelligence Scale for Children-III, Scandinavian norms	Present fidgety movements plus abnormal^1^ concurrent movements related to present fidgety movements plus normal^2^ concurrent movements related to total *IQ* < 85 vs. total *IQ* ≥ 85	Sensitivity: 90%(95% CI: 60–98%)Specificity: 58%(95% CI: 39–76%)PPV: 53%(95% CI: 31–74%)NPV: 93%(95% CI: 69–99%)
Grunewaldt et al., 2014	*N* = 64*n* = 31 with ELBW, mean gestational age = 26.1 weeks (*SD* = 1.8) *n* = 33 controls born at term	**10 years** Wechsler Intelligence Scale for Children-III, Scandinavian norms;Stroop Color Word;Tower of London Test;Trail-Making Test	Not given	Not given

*^1^Movements other than fidgety movements appear to be monotonous and/or jerky and/or stiff; ^2^all movements are carried out smoothly and fluently (according to Einspieler et al., 2004, p. 26). CI, confidence interval; IQ, intelligence quotient; MDI, mental developmental index; NPV, negative predictive value; PPV, positive predictive value; SD, standard deviation.*

## The Link between Early Normalization of GMs and Cognitive Development

Many preterm infants – and ELBW infants in particular – show poor repertoire GMs during their first few days of life (de Vries et al., 2008; de Vries and Bos, 2010). Some of them normalize within a few weeks (de Vries and Bos, 2010), whereas others only normalize around term-equivalent age or later (Bruggink et al., 2010; de Vries and Bos, 2011). Some infants exhibit poor repertoire GMs until they reach the age of fidgety movements, i.e., 3–5 months. If fidgety movements are present and normal, the motor outcome will be normal, whereas absent fidgety movements point to a neurodevelopmental dysfunction (Prechtl et al., 1997; Nakajima et al., 2006). Bruggink et al. (2010) were the first to associate the age of GM normalization with later cognition. They longitudinally assessed the GMs of 60 preterm infants from birth to early infancy and compared their findings to the results of the Wechsler Intelligence Scale for Children, third edition, applied at age 7–11 years. IQs – both verbal and performance – were around 100, regardless of whether GMs were normal from the beginning or normalized before term. However, abnormal (i.e., poor repertoire) GMs that persisted until 8 weeks after term were related to IQs almost 1 *SD* below the mean (median total *IQ* = 87; median verbal *IQ* = 88; median performance *IQ* = 88). School performance was also related to the quality of GMs. The percentage of children who had to repeat a class or attended special education was higher where GMs did not normalize by 8 weeks after term (Bruggink et al., 2010).

Two other studies confirmed the results of Bruggink et al. (2010), although their outcome assessments were carried out at a much lower age. Beccaria et al. (2012) assessed the development of preterm-born children aged 2 years by means of the Griffiths Scales of Mental Development (**Table 1**) and compared the DQs of children who had shown normal writhing GMs with the DQs of children who had exhibited poor repertoire GMs at 1 month after term. Where GMs were still poor repertoire at 1 month after term, the DQ was 11 points lower (mean = 97, *SD* = 12) than in children with normal GMs at 1 month (mean = 108, *SD* = 11; *p* < 0.01). Only the sub-scales “hearing and speech,” “eye and hand coordination,” and “performance” contributed to these results, whereas the sub-scales “locomotion” and “personal/social” did not. Similar results were found for a smaller sample of Slovenian preterm infants: 15 in 26 preterm infants still had poor repertoire GMs 1 month after term. Their MDI assessed at 2–3 years with the Bayley Scales of Infant Development, second edition, was, on average, eight points lower (mean = 95, *SD* = 11) than that of children with normal writhing GMs (mean = 103, *SD* = 9; *p* < 0.05). Children with cramped-synchronized writhing GMs after term age were more likely to develop motor problems and had MDIs which indicated mental developmental delay (mean = 76, *SD* = 22). Spittle et al. (2013) obtained a slightly different result. In their sample of very preterm-born children (i.e., at <30 weeks’ gestation) the cognitive score of the Bayley Scales of Infant and Toddler Development, third edition, assessed at age 2, was only an average of five points lower in children with abnormal GMs at 1 month after term (mean = 96.6, *SD* = 13.2) than that of children who had had normal GMs (mean = 101.4, *SD* = 11.7; *p* = 0.06). Assessed at 4 years, the same children exhibited no difference whatsoever: general reasoning and conceptual abilities bore no relation to the GMs assessed at 1 month after term.

To sum up, the association between writhing GMs and cognitive development is variable, with increasing evidence that abnormal (poor repertoire) GMs – if still present after term – are associated with an MDI/IQ 5–13 points lower than that of children whose writhing GMs were normal. Defining an MDI or *IQ* < 85 as a moderately impaired cognitive outcome, the sensitivity values reached 67–100%, while the specificity values ranged from 28–71% (**Table 1**; Bruggink et al., 2010; Kodric et al., 2010; Spittle et al., 2013).

## Fidgety Movements May not Predict Cognitive Development, but Concurrent Movements And postures Do

Most of the eligible studies reported that fidgety movements were not related to cognitive development (Butcher et al., 2009; Fjørtoft et al., 2013; Grunewaldt et al., 2014). Only Spittle et al. (2013) found in their study on 94 infants born at <30 weeks’ gestation that the Bayley-III cognition score assessed at 2 years was, on average, eight points higher in children who had had normal fidgety movements (mean = 100.4, *SD* = 10.8) than in children with absent or abnormal fidgety movements (mean = 92, *SD* = 17.6; *p* < 0.05). The difference between the two groups was even more significant at the 4-year-assessment: the cognitive score of children who had shown normal fidgety movements at 3–5 months was, on average, 14 points higher (mean = 99.8, *SD* = 13.4) than that of children with abnormal or absent fidgety movements (mean = 85.5, *SD* = 18.3; *p* < 0.01; Spittle et al., 2013).

Other authors have related early abnormalities in the posture or the overall movement character to sub-optimal cognition at school age. Butcher et al. (2009) were the first to investigate whether the quality of movements at 3–5 months could predict cognitive performance at school age. They studied 65 children born preterm and found that the number of normal postural patterns displayed between 11 and 16 weeks after term contributed significantly to the prediction of total and verbal IQs, and almost significantly to that of performance IQs. Certain postural patterns such as whether or not infants kept the head in the midline, had a symmetric body posture, or showed various finger postures might reflect the increase of activity levels in several cortical areas as well as in the cerebellum and basal ganglia at 3 months (Chugani et al., 1987). Visual and manual exploration becomes more active and better coordinated by that age (Prechtl, 1986; Einspieler et al., 2004). Independent and variable finger movements facilitate object manipulation and exploration, supplementing visual with extero- and proprioceptive input (Rosenbaum et al., 2012).

Fjørtoft et al. (2013) found that the overall movement character (smooth and fluent vs. monotonous, jerky and/or stiff) at 3–5 months predicted the children’s IQ at age 10 years with a sensitivity of 90% and a specificity of 58% (**Table 2**). Similar findings were reported by Grunewaldt et al. (2014) for a group of 31 ELBW infants. Those 20 ELBW infants who did not develop cerebral palsy had normal fidgety movements, but only nine of them had a smooth and fluent movement character. The remaining 11 children with monotonous, jerky, and/or stiff movements in early infancy developed a lower working memory capacity and lower processing speed, but showed no differences with regard to total IQ scores. On magnetic resonance imaging, they had a lower volume of cerebral white matter volume at 3–5 months than children with normal movements. Comparing clinical characteristics, the only difference between the groups was that infants with monotonous, jerky and/or stiff movements were more often small-for-gestational-age singletons than infants with a smooth and fluent movement character. The authors speculated that perhaps fetal growth restriction including the brain had caused the reduced cognitive functioning at school age (Grunewaldt et al., 2014).

## Limitations of the Studies

Almost none of the authors distinguished between the sub-categories of abnormal writhing GMs. Instead, they labeled them as one “abnormal” category, while mentioning that the majority of abnormal GMs were scored as poor repertoire. Spittle et al. (2013) also pooled abnormal and absent/sporadic fidgety movements into “abnormal GMs at 3 months.” More details on abnormal GMs at 3 months would substantially add to our understanding of abnormal fidgety movements, whose predictive value is not yet clear (Prechtl et al., 1997; Einspieler et al., 2007; Bruggink et al., 2008; Yuge et al., 2011). None of the studies focused on individual developmental trajectories. The question remains: which abnormal writhing movements lead to which peculiarities at the age of fidgety movements? Do infants with poor repertoire GMs also show a monotonous movement pattern at 3–5 months? Future studies need to shed light on specific individual developmental trajectories of preterm infants and relate them to the children’s later cognitive performance.

Another common flaw is that cognitive dysfunction tends to be pooled with motor problems. A considerable number of children studied by Fjørtoft et al. (2013) had both balance problems and a poor cognitive outcome. It is therefore difficult to answer conclusively if there are specific GM markers for cognitive dysfunction.

One final deficiency is the small number of studies on the topic. While only seven studies met the inclusion criteria for our review, we believe that the globally increasing clinical and scientific application of the GMA will bring some remarkable new findings in the very near future. One development that promises to boost activity in the field is the use of smartphone-based applications.

## Conclusion

The above-mentioned studies on the GMA’s predictive value for cognitive development suggest the following: clinicians should be aware that abnormal movements are not only associated with motor impairments but also with potential adverse outcomes in other developmental domains. Abnormal GMs beyond term age and monotonous and jerky movements as well as postural abnormalities at 3–5 months might indicate a high risk for a subsequent cognitive dysfunction. A monotonous motor repertoire during these early months of development might have an adverse effect on the infants’ abilities to interact with their environment (Hadders-Algra, 2000). Further and more comprehensive research is needed, although the existing body of literature makes a strong case for early intervention services and follow-up examination to improve the long-term cognitive development of children born preterm.

## Author Contributions

CE: contributed substantially to the conception of the work, the acquisition, and interpretation; drafted the work and approved the version to be published; agreed to be accountable for all aspects of the work in ensuring that questions related to the accuracy or integrity of any part of the work are appropriately investigated and resolved. AB, ML, and PM: contributed substantially to the design of the work, and interpretation; revised the first draft critically for important intellectual content and approved the version to be published; agreed to be accountable for all aspects of the work in ensuring that questions related to the accuracy or integrity of any part of the work are appropriately investigated and resolved.

## Conflict of Interest Statement

The authors declare that the research was conducted in the absence of any commercial or financial relationships that could be construed as a potential conflict of interest.

## Abbreviations

DQ: developmental quotient
ELBW: extremely low birth weight
GMs: general movements
GMA: general movement assessment
IQ: intelligence quotient
MDI: mental developmental index
SD: standard deviation.
